# Efficacy and safety of a modified lateral lumbar interbody fusion in L4-5 lumbar degenerative diseases compared with traditional XLIF and OLIF: a retrospective cohort study of 156 cases

**DOI:** 10.1186/s12891-022-05138-7

**Published:** 2022-03-07

**Authors:** Jiaqi Li, Yapeng Sun, Lei Guo, Fei Zhang, Wenyuan Ding, Wei Zhang

**Affiliations:** Department of Spinal Surgery, The Third Hospital of Hebei Medical University, Shijiazhuang, 050000 Hebei Province China

**Keywords:** Extreme lateral interbody fusion, Oblique lumbar interbody fusion, Lateral lumbar interbody fusion, Complication, Modified procedure

## Abstract

**Background:**

The authors designed a modified lateral lumbar interbody fusion (LLIF) procedure named as XOLIF and compared the efficacy and safety with traditional LLIF procedures.

**Methods:**

Patients were divided into XLIF, OLIF, and XOLIF group according to the surgical approach. Cases of psoas major and vascular space stenosis, psoas major muscle elevation, psoas major muscle hypertrophy, and high iliac crest were recorded. Basic information, composition ratio of specific cases, Visual analog scale (VAS), Oswestry Disability Index (ODI), interbody fusion rate and complications were compared between the 3 groups.

**Results:**

The study included 156 cases of L4-5 LLIF. There was no statistical difference in age, gender, BMI among the three groups. Cases with stenosis between psoas muscle and artery accounted for 11.8 and 18.4% of the XLIF and XOLIF group, respectively, while no case of this type had undergone OLIF surgery, the difference was statistically significant (*P* < 0.05). The proportions of high iliac crest cases in the OLIF and XOLIF group were 12.5 and 18.4%, respectively, while the XLIF group with vertical approach is not suitable for cases with high iliac crest. The postoperative VAS and ODI of the three groups were significantly improved compared with those before operation. There were 51 cases (32.7%) of complications including 21cases in XLIF group, 20 cases in OLIF Group and 10 cases in XOLIF group. XOLIF group has more advantages in reducing lumbar plexus injury and the risk of vascular injury.

**Conclusions:**

XOLIF showed good clinical efficacy and technical advantages with a low incidence of intraoperative and postoperative complications, especially in the specific cases.

## Background

Extreme lateral interbody fusion (XLIF) technique was first introduced by Pimenta and Taylor in 2006 as an alternative to traditional anterior lumbar interbody fusion [1]. The technique enables access to the spine laterally via the retroperitoneal corridor by splitting the fibers of the psoas muscle longitudinally. Over the past decade, XLIF has established itself as an effective means and adjunct when treating an array of spinal pathology and are increasingly being utilized as a minimally invasive approach to treat an array of spinal pathology [2, 3].

Although specialized equipment and neuromonitoring techniques were used to maximize the safety and reproducibility, XLIF still pose a unique risk to the lumbosacral plexus housed within the psoas muscle, because this approach is essentially a transpsoas approach [4, 5]. Injuries of this sort are referred to as lumbar plexopathies and are reported to occur as high as 0-75%, especially at L4-5 Level [6]. To avoid this complication, several technical innovations have been proposed by experts and produced good clinical outcomes [7, 8]. Most of these innovations are based on direct visualization and special designed distractor, and this makes it possible to perform a XLIF procedure without neuromonitioring, which, to our known, is a common and well accepted practice in some era in China.

Previously, we proposed a modified XLIF procedure based on clinical anatomy research results published before [9, 10]. We split psoas major muscle at the anterior 1/3 point of intervertebral space which is relatively nerve free area and put in a 3 blades retractor vertically. Early clinical series study proves that this procedure is safe and easy to master compare to traditional XLIF procedure which split poses muscle at midpoint of intervertebral space usually. But in later practice, we find that it is still quite difficult to manipulate a 3 blades retractor to a vertical position when performing L45 XLIF procedure for heavy patients with strong psoas major muscle and high iliac crest. The main reason for this may be as follows: 1) Direct blockage by high crest iliac 2) Tightness of strong psoas major muscle due to a jack-knife position despite hip and knee joint are flexed. 3) Three blades interfere each other when retractor is expanded. When 3 blades retractor can not be manipulated to a vertical position, cage will be inserted in a lean direction. Until last year, we have 2 revision cases due to nerve compression by mal-positioned cage. Failure of these cases reminded us that further technical innovation should be explored.

As we all known, oblique lumbar interbody fusion (OLIF) technique is another popular lateral fusion procedure through prepsoas approach, one of the advantages of OLIF is less injury to the lumbosacral plexus. Based on that we designed a new lateral lumbar interbody fusion (LLIF) procedure characterized as more anterior incision compare to XLIF, splitting psoas muscle at its anterior edge, using 2 blades retractor and put it in obliquely, inserting cage vertically finally. In fact, this innovation is a combination technique of traditional OLIF and XLIF, so we named it Extreme-oblique lumbar interbody fusion (XOLIF). In this clinical investigation, we try to find out whether this combination technique can be more effective than traditional XLIF and OLIF technique alone and avoid some inherence pitfalls of these traditional technique.

## Methods

**Fig. 1:**
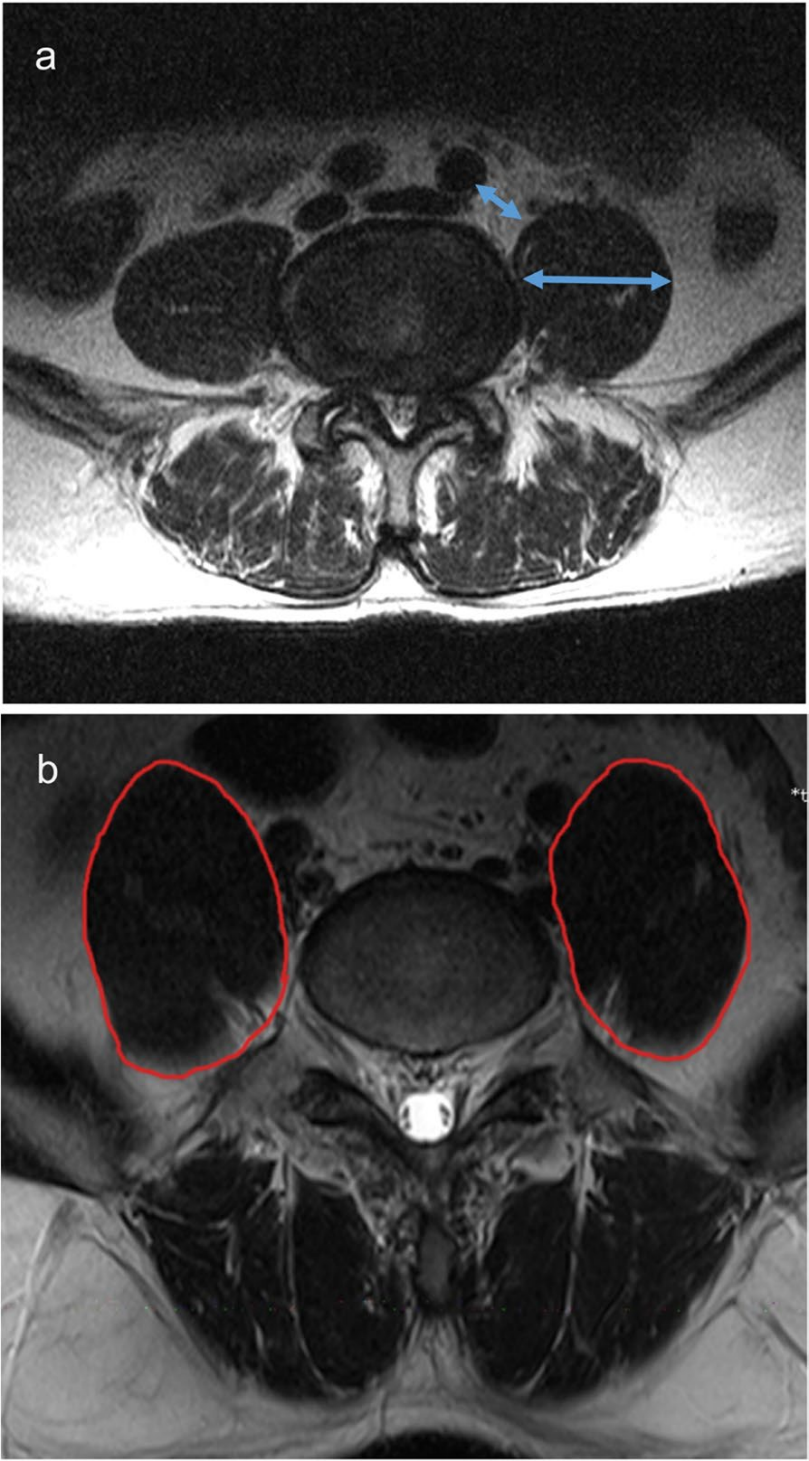
**a** Measurement of the size of the space between psoas major muscle and large artery, and the thickness of psoas major muscle by preoperative axial T2-weighted MRI image at surgical segment. **b** Typical psoas major muscle elevation from axial T2-weighted MRI images at surgical segment

The study was approved by the hospital ethics committee, and all patient data collected were informed in advance. Cases of LLIF surgery were collected from May 2017 to December 2019 in our center. Cases were divided into XLIF group, OLIF group, and XOLIF group according to the surgical approach. XOLIF was mostly used in cases of psoas major and vascular space stenosis, psoas major muscle elevation, psoas major muscle hypertrophy, and high iliac crest. The size of the space between psoas major muscle and large artery, and the thickness of psoas major muscle was measured by preoperative MRI (Fig. 1a). We defined the size of the space between psoas major muscle and large artery to be less than 2 mm as stenosis, and the psoas major muscle thickness greater than 5 cm as hypertrophy. The elevation of psoas major referred to the significant elevation of the ventral portion of the psoas major over its medial artery (Fig. 1b). High iliac crest was defined as the highest point of the iliac crest being significantly higher than the surgical intervertebral space on the lumbar anterolateral radiograph.

### Inclusion and exclusion criteria

Inclusion criteria: 1. Lumbar spinal stenosis, grade 1 spondylolisthesis, lumbar discogenic back pains; 2. Conservative treatment was ineffective for more than 3 months; 3. Patients undergo L45 single-segment LLIF; 4. Follow-up for more than 1 year.

Exclusion criteria: 1. Lumbar spine tumors, infectious diseases; 2. Lumbar spine surgery history at the same level; 3. Abdominal and retroperitoneal surgical history; 4. Incomplete follow-up data for 3, 6, and 12 months after surgery.

The basic information of age, sex, Body mass index (BMI) and operative segment were recorded. The cage we used in the study was Zimmer, Johnson Oracle or Medtronic Clydesdale cage. Not all cases included in this study were examined by dual energy X-ray or QCT, so the data were not counted. The dual energy X-ray examination was mostly carried out for patients older than 50 years old. For other patients, the bone mass of patients was preliminarily evaluated according to X-ray and CT of the lumbar spine. We used the above methods to judge bone health of patients. In this study, the internal fixation method was determined by comprehensively considering the patient’s age, BMI, bone mass, disease type and economic conditions of patients. The internal fixation method included posterior pedicle screw fixation, lateral fixation and stand-alone. Lateral fixation included self-stabilized anchor plate (Zimmer) and lateral plate (Doudle Medical). Cases of psoas major and vascular space stenosis, psoas major muscle elevation, psoas major muscle hypertrophy, and high iliac crest were recorded. Visual analog scale (VAS) was used to evaluate the lumbocrural pain, and Oswestry disability index (ODI) was used to evaluate the functional recovery. Postoperative CT scan was used to evaluate interbody fusion. Solid intervertebral fusion was defined as the bridging bone trabeculae visible on two consecutive CT planes.

### XO-LIF procedure

#### Patient preparation

**Fig. 2:**
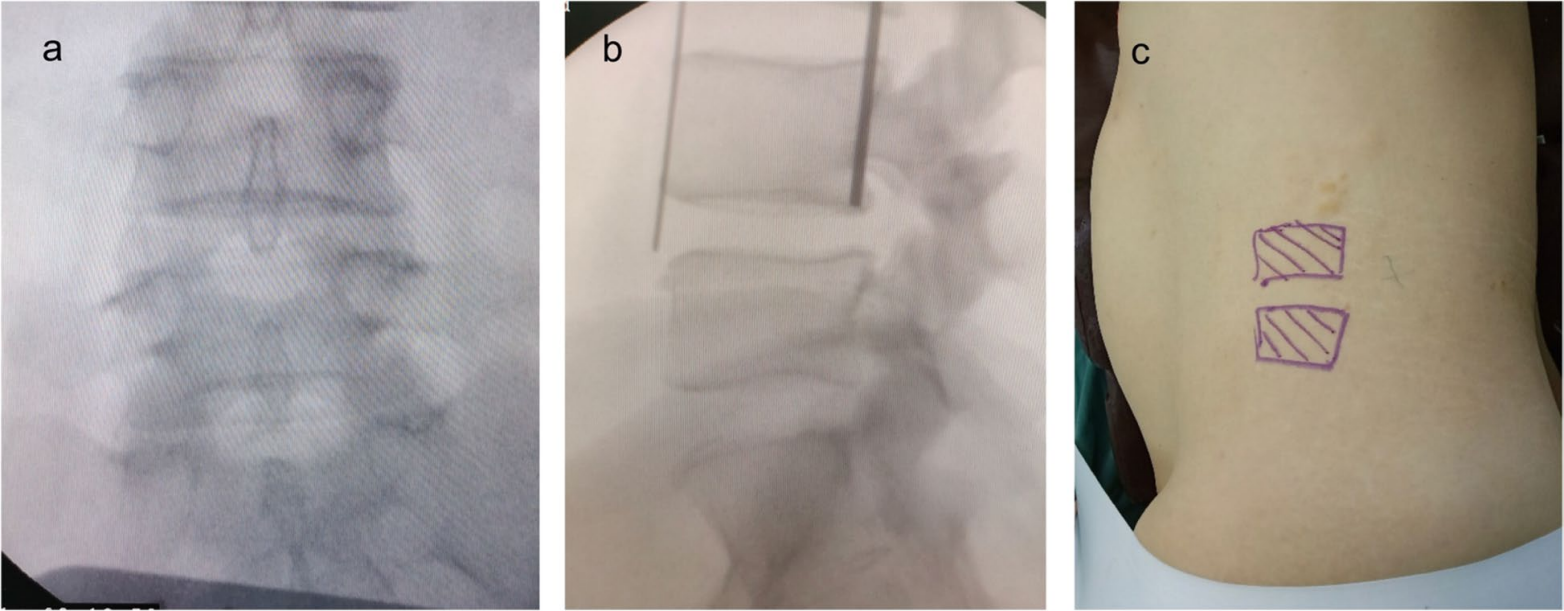
The operative segment is confirmed to be in absolute lateral position under fluoroscopy and the surface location is marked. **a** The spinous process is located in the center between two pedicles in anteroposterior X-ray. **b** The upper endplate of caudal vertebrae overlaps in a line and the affected disc space is marked with 2 K-wires in lateral X-ray. **c** The surface location of the affected disc space is marked on the patient’s lateral side

Patients lie on a true 90° left or right lateral decubitus position after general anesthesia, bend hips and knees, without making the break of the table between the iliac crest and greater trochanter for side bending. The operative segment is confirmed to be in absolute lateral position under fluoroscopy, and the surface location of the affected disc space is marked on the patient’s lateral side (Fig. 2).

#### Incision and retroperitoneal access

A 4 cm incision is made at the anterior edge of the vertebral body longitudinally. In cases of high iliac crest, the incision was moved ventrally to the anterior edge of the iliac crest, avoiding iliac crest. Incision of subcutaneous fat and blunt separation of the abdominal wall muscle to the retroperitoneal space is performed.

Touch the psoas muscle with finger and separate the retroperitoneal fat. Use a retractor to retract the retroperitoneal fat to the dorsal side to expose the psoas muscle surface. The fat tissue on the surface of psoas major muscle is obtuse separated with “pignut” under direct vision, and the anterior edge of psoas major muscle is located.

#### Trans-psoas access at anterior edge of psoas major muscle

**Fig. 3:**
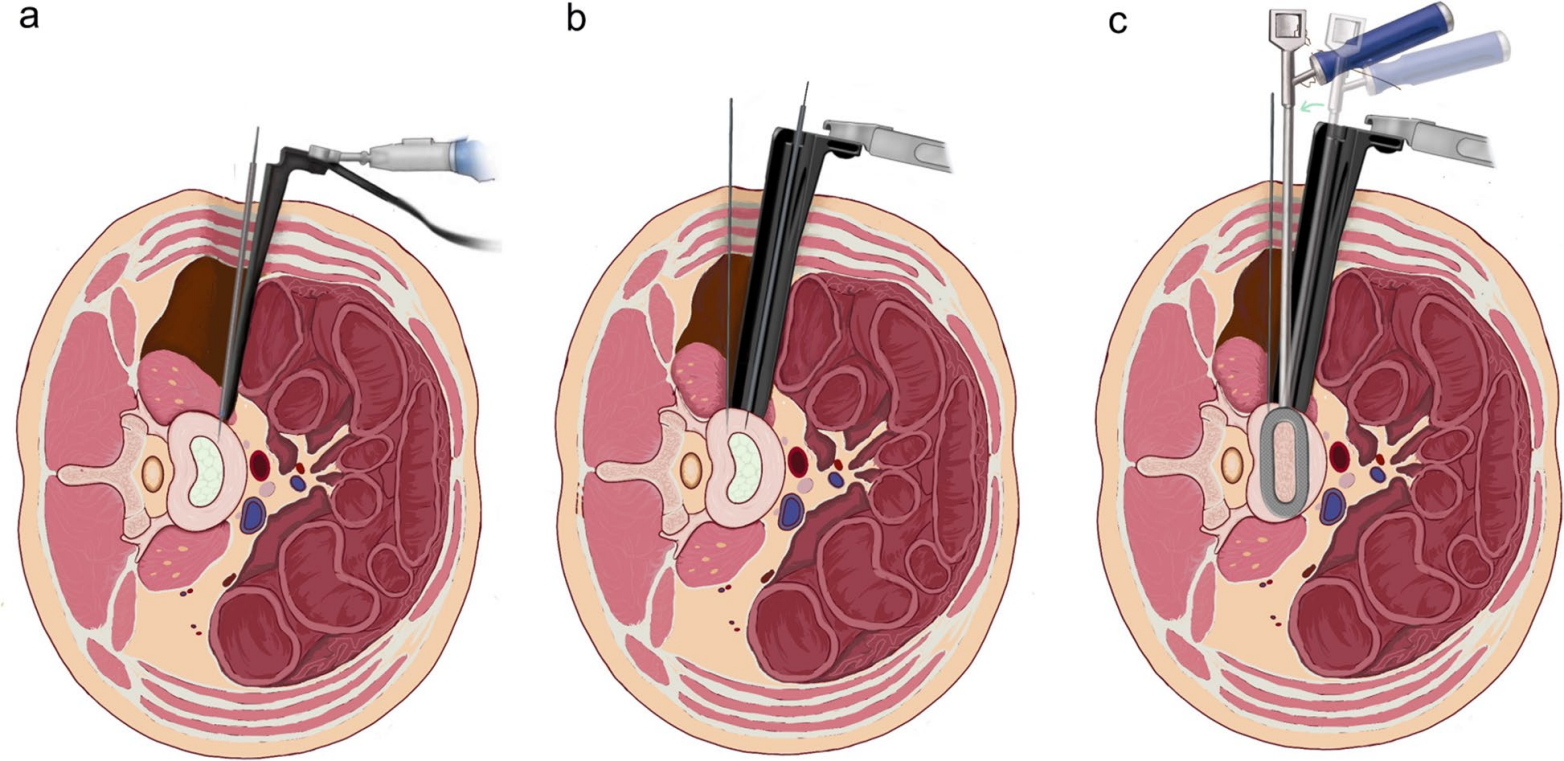
Schematic diagram of the intraoperative procedure. **a** Initial position with a guide rod after dissecting aponeurosis of anterior edge of psoas major muscle under direct vision. **b** Expose the operation area with 2 blades retractor and Kirschner wire. **c** Implant a cage vertically after discectomy

The aponeurosis of anterior edge of psoas major muscle was dissected. The fibers of psoas major muscle were splitted behind the aponeurosis to the intervertebral space longitudinally in the next, and a guide rod is used to guide the implantation of a Kirschner wire (Fig. 3a).

After the affected disc space is confirmed by lateral fluoroscopy, 2 blades retractor is placed obliquely along the guide rod, and fixed with a free arm. The operation area is initially revealed after the blades of retractor are expanded longitudinally. At this point, the hypertrophic psoas muscle still blocks the operation area. The psoas muscle is pushed back with a periosteal dissector, and two 3.0 mm-diameter Kirschner-wires are inserted into the cranial and caudal side of the vertebral body near the endplate, so the psoas muscle is pulled back by the blocking action of Kirschner wire. At the same time, a 1.5 mm-diameter Kirschner wire with a guide rod is inserted into the center of the lateral intervertebral space (Fig. 3b).

#### Discectomy and cage placement

The guide rod tip is located under lateral fluoroscopy to confirm the position of the intervertebral disc incision, which can avoid the incision being too ventral or dorsal. The disc is then incised and the cartilage endplates are scraped off. After rinsing with normal saline, a cage filled with allogeneic bone paste and bone morphogenetic protein (BMP) is implanted vertically (Fig. 3c). After adequate hemostasis, we suture the incision layer by layer without drainage.

### Statistical analysis

We use SPSS 23.0 for statistical analysis. Measurement data was expressed as mean ± standard deviation. When measurement data conformed to normal distribution and the test for homogeneity of variance was homogeneous, the analysis of variance was used for comparison among groups; otherwise, Kruskal Wallis test was used. Measurement data within each group was compared at different time points using single-factor repeated measures analysis of variance for statistical analysis. Count data was reported as a percentage, and Chi-square test or Fisher test was used for comparison among groups and within groups. *P* < 0.05 indicated that there was a statistical difference.

## Results

The study included 156 cases of LLIF, including 51 cases in the XLIF group, 56 cases in the OLIF group, and 49 cases in the XOLIF group. In XLIF group, there were 30 cases of lumbar spinal stenosis, 17 cases of lumbar spondylolisthesis and 4 cases of discogenic low back pain. In OLIF group, there were 33 cases of lumbar spinal stenosis, 20 cases of lumbar spondylolisthesis and 3 cases of discogenic low back pain. In XOLIF group, there were 28 cases of lumbar spinal stenosis, 19 cases of lumbar spondylolisthesis and 2 cases of discogenic low back pain. There was no statistical difference in age, gender and BMI among the three groups (Table 1). T score or QCT values were not counted in this study. In XLIF group, there were 21 cases of posterior pedicle screw fixation, 12 cases of lateral fixation and 18 cases of stand-alone. There were 27 cases of posterior pedicle screw fixation, 9 cases of lateral fixation and 20 cases of stand-alone in OLIF group. There were 24 cases of posterior pedicle screw fixation, 8 cases of lateral fixation and 17 cases of stand-alone in XOLIF group. There was no significant difference in methods of internal fixation between groups (*P* > 0.05). The average follow-up time of the XLIF group and OLIF group was 24.08 months and 25.30 months, respectively. The traditional XLIF and OLIF procedures were carried out in our center during the same period, and there was no statistical difference in the follow-up time. While XOLIF is our technical improvement after a period of application of traditional XLIF and OLIF, so that, average follow-up of this group was 18.84 months, which was shorter than that of the traditional lateral surgery group (*P* < 0.001). The average operation time (XLIF group, 84.98 min ± 15.05; OLIF group, 85.41 min ± 14.70; XOLIF group, 81.96 min ± 10.73) and intraoperative average blood loss (XLIF group, 63.73 ml ± 25.75; OLIF group, 68.75 ml ± 35.27; XOLIF group, 65.10 ml ± 24.67) of the three groups had no statistical difference (*P* > 0.05, Table 1).

**Table 1:** Basic information of group XLIF, OLIF and XOLIF

Values	XLIF	OLIF	XOLIF	*P* (2-tailed)
Age (year)	56.74 ± 12.94	54.82 ± 12.88	53.53 ± 13.78	0.537^$^
Sex (male/female)	19/32	25/31	16/33	0.442^&^
BMI (kg/m^2^)	25.22 ± 2.79	24.75 ± 3.29	25.06 ± 2.71	0.707^#^
Operation time (min)	84.98 ± 15.05	85.41 ± 14.70	81.96 ± 10.73	0.625^$^
Intraoperative blood loss (ml)	63.73 ± 25.75	68.75 ± 35.27	65.10 ± 24.67	0.831^$^
Follow-up time (month)	24.08 ± 6.56_a_	25.30 ± 6.54_a_	18.84 ± 3.28_b_	< 0.001^#^

*XLIF* indicated extreme lateral interbody fusion, *OLIF* oblique lumbar interbody fusion; ^$^, Kruskal Wallis test; ^&^, Pearson Chi-square test; #, Analysis of variance; Multiple comparisons were used bonferroni method, and at least one identical subscript letter denotes do not differ significantly from each other

Table 2 showed the composition ratio of cases with stenosis between psoas muscle and artery, psoas major muscle elevation, psoas major muscle hypertrophy, and high iliac crest in each groups. Cases with stenosis between psoas muscle and artery accounted for 11.8 and 18.4% of the XLIF group and XOLIF group, respectively, and there was no significant difference in the proportion of this type of cases between the two groups; while no case of this type had undergone OLIF surgery, the difference was statistically significant (*P* < 0.05). There was no significant difference in the proportion of cases with psoas major hypertrophy in three groups (*P* > 0.05). The XLIF group, OLIF group and XOLIF group included 4, 0, and 8 cases of elevated psoas major muscles, respectively, and the composition ratio of the three groups was statistically different (*P* < 0.05). The proportions of high iliac crest cases in the OLIF group and XOLIF group were 12.5 and 18.4%, respectively, while the traditional XLIF procedure with vertical approach is not suitable for cases with high iliac crest. The composition ratio of high iliac crest cases in three groups was statistically different (P < 0.05).

**Table 2:** The condition of iliac crest and psoas major of group XLIF, OLIF and XOLIF

	XLIF	OLIF	XOLIF	*P* (2-tailed)
Number of cases	51	56	49	
Stenosis between psoas muscle and artery	6 (11.8%)_a_	0 (0%)_b_	9 (18.4%)_a_	0.001^&^
Psoas major muscle hypertrophy	7 (13.7%)_a_	8 (14.3%)_a_	12 (24.5%)_a_	0.275^&^
Psoas major muscle elevation	4 (7.8%)_a_	0 (0%)_b_	8 (16.3%)_a_	0.001^&^
High iliac crest	0(0%)_a_	7 (12.5%)_b_	9 (18.4%)_b_	0.008^&^

*XLIF* indicated extreme lateral interbody fusion, *OLIF* oblique lumbar interbody fusion; ^&^, Pearson Chi-square test; Multiple comparisons were used bonferroni method, and at least one identical subscript letter denotes do not differ significantly from each other

**Fig. 4:**
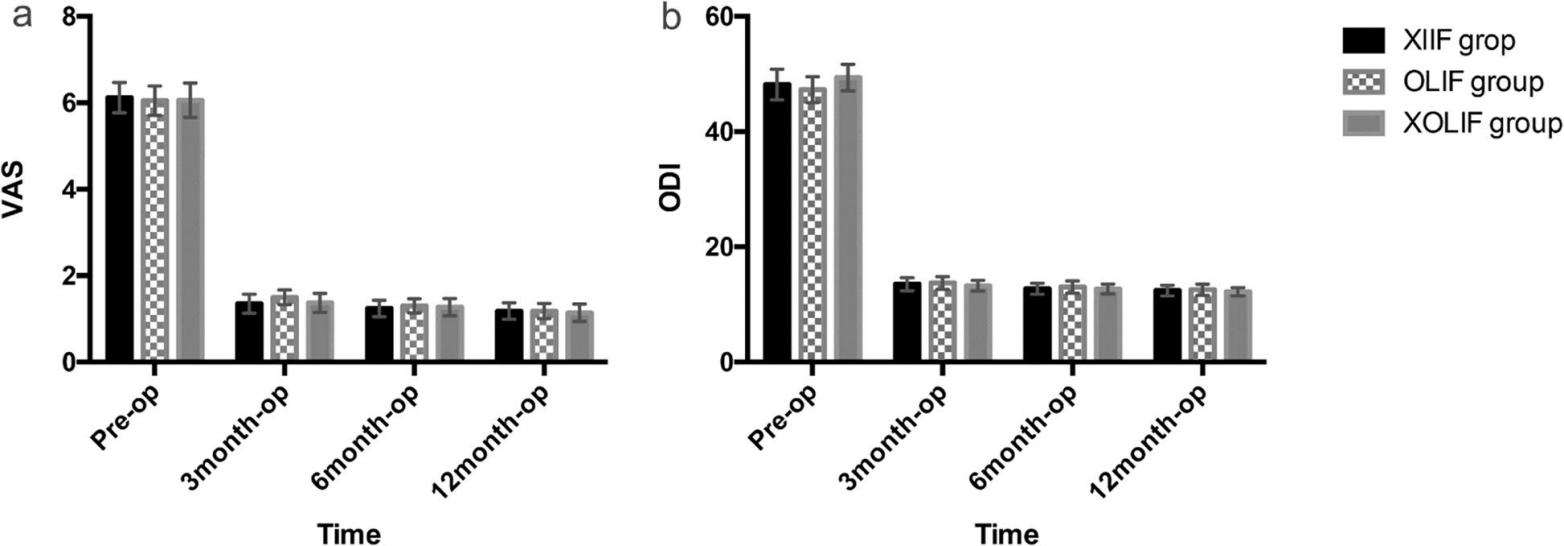
Postoperative VAS (**a**) and ODI (**b**) were significantly reduced compared with those of preoperation

**Fig. 5:**
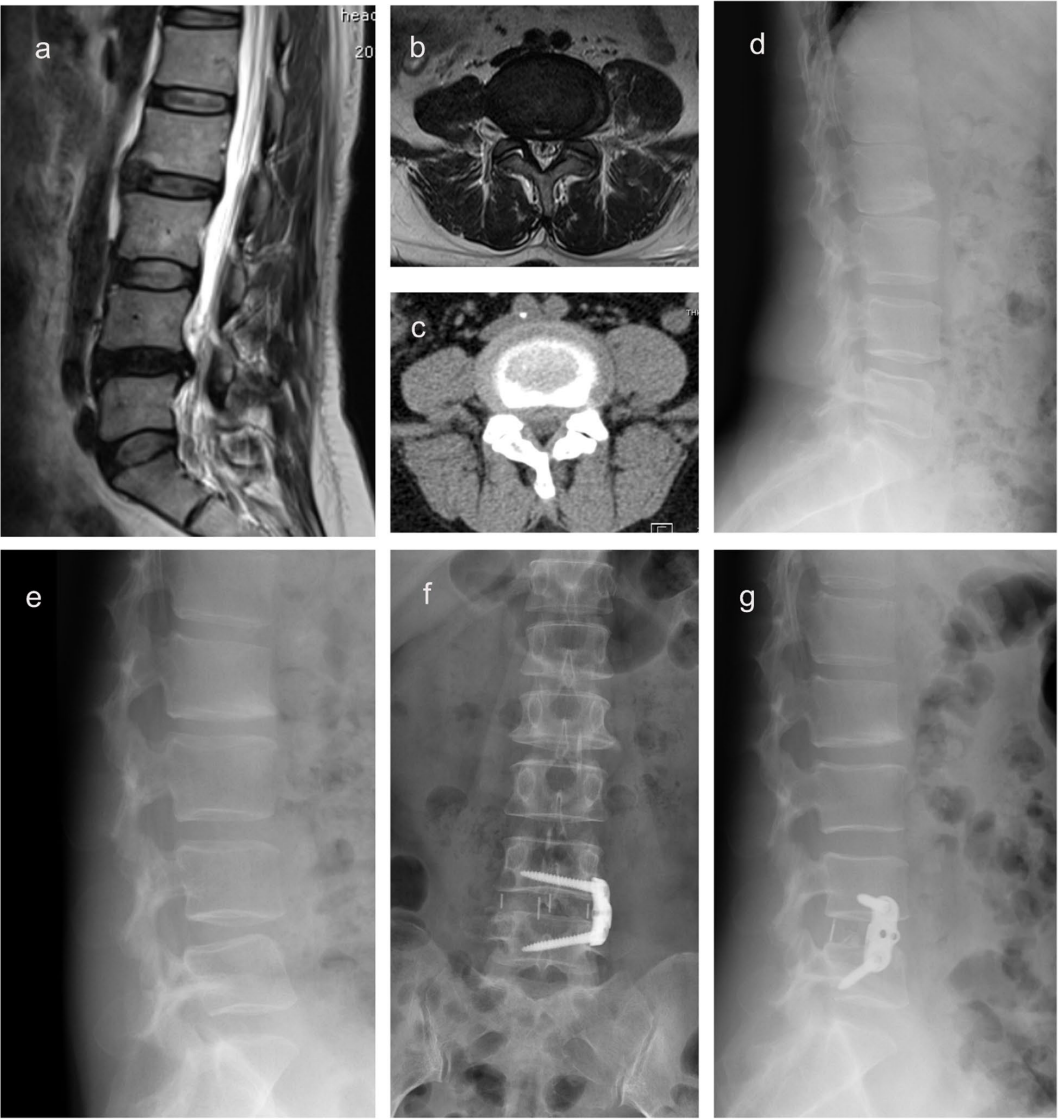
Preoperative and postoperative radiographs of a case with lumbar spinal stenosis. **a** Preoperative sagittal T2-weighted MRI image. **b** Preoperative axial T2-weighted MRI image indicated lumbar stenosis at L4-5. **c** Preoperative axial CT showed that lumbar stenosis at L4-5. **d**, **e** Preoperative flexion-extension stress lateral radiographs. **f**, **g** Anteroposterior X-ray of lumbar spine after XLIF

The VAS and ODI of the three groups before operation, 3, 6, and 12 months after operation were shown in Table 3. In the XLIF group, the mean ± standard deviation of VAS and ODI before operation was 6.12 ± 1.24 and 48.18 ± 9.39, respectively; VAS and ODI at the last follow-up was 1.18 ± 0.68 and 12.41 ± 3.31, respectively. In the OLIF group, the mean ± standard deviation of VAS and ODI before operation was 6.05 ± 1.26 and 47.27 ± 8.46, respectively; VAS and ODI at the last follow-up was 1.18 ± 0.66 and 12.55 ± 3.55, respectively. In the XOLIF group, the mean ± standard deviation of VAS and ODI before operation were 6.06 ± 1.39 and 49.37 ± 8.03, respectively; VAS and ODI at the last follow-up were 1.14 ± 0.71 and 12.20 ± 2.54, respectively. The VAS and ODI of the three groups in each postoperative period were significantly improved compared with those before operation (*P* < 0.05, Fig. 4). In both XLIF group and OLIF group, VAS significantly decreased at 12 months after operation compared with 3 months after operation, and ODI significantly decreased at 6 and 12 months after operation compared with 3 months after operation (*P* < 0.05). In XOLIF group, ODI significantly decreased at 12 months after operation compared with 3 months after operation (*P* < 0.05). The interbody fusion rates evaluated by CT scan in the XLIF group, OLIF group, and XOLIF group at 12 months after surgery were 92.1, 91.0, and 93.8%, respectively, and the difference was of no statistical significance (*P* > 0.05). There was no significant difference in preoperative and postoperative radiographs between the groups. Figure 5 showed preoperative and postoperative radiographs of a case.

**Table 3:** Clinical efficacy and intervertebral fusion rate

Values		Pre-op	3 month	6 month	12 month	*P* (2-tailed)
	XLIF	6.12 ± 1.24_a_	1.35 ± 0.77_b_	1.24 ± 0.68_bc_	1.18 ± 0.68_c_	< 0.001^†^
VAS	OLIF	6.05 ± 1.26_a_	1.50 ± 0.63_b_	1.30 ± 0.60_bc_	1.18 ± 0.66_c_	< 0.001^†^
	XOLIF	6.06 ± 1.39_a_	1.37 ± 0.77_b_	1.27 ± 0.70_b_	1.14 ± 0.71_b_	< 0.001^†^
	*p*	0.912^$^	0.439^$^	0.905^$^	0.967^$^	–
	XLIF	48.18 ± 9.39_a_	13.51 ± 4.02_b_	12.73 ± 3.39_c_	12.41 ± 3.31_c_	< 0.001^†^
ODI (100%)	OLIF	47.27 ± 8.46_a_	13.73 ± 4.10_b_	13.02 ± 3.88_c_	12.55 ± 3.55_c_	< 0.001^†^
	XOLIF	49.37 ± 8.03_a_	13.24 ± 3.24_b_	12.69 ± 2.97_bc_	12.20 ± 2.54_c_	< 0.001^†^
	*p*	0.464^#^	0.859^$^	0.974^$^	0.969^$^	–
	XLIF	–	–	–	47/4	–
Fusion (n. yes/no)	OLIF	–	–	–	51/5	–
	XOLIF	–	–	–	46/3	–
	*p*	–	–	–	0.861	–

*XLIF* indicated Extreme lateral interbody fusion, *OLIF* oblique lumbar interbody fusion, *VAS* Visual analogue scale, *ODI* Oswestry disability index; †, repeated measurement analysis of variance; ^$^, Kruskal Wallis test; ^#^, Analysis of variance; Multiple comparisons of each variable at different time points were used bonferroni method, and at least one identical subscript letter denotes do not differ significantly from each other

Perioperative complications were shown in Table 4. Complications were found in 38 of 156 patients (16 in XLIF group, 14 in OLIF Group and 8 in XOLIF Group). There were 51 cases (32.7%) of complications including 21cases in XLIF group, 20 cases in OLIF Group and 10 cases in XOLIF group.

**Table 4:** Intraoperative and postoperative complications of XLIF, OLIF and XOLIF

Complication	XLIF	OLIF	XOLIF
**Intraoperative cases (%)**	–	–	–
Segmental lumbar artery	0	1 (1.8)	0
Ascending lumbar vein	0	1 (1.8)	0
Iliac vein	0	1 (1.8)	0
Other vessels	0	0	0
Ureteral injury	0	0	0
Peritoneal laceration	0	1 (1.8)	0
Other organ injury	0	0	0
Endplate injury	3 (5.9)	2 (3.6)	2 (4.1)
**Postoperative cases (%)**	–	–	–
Contralateral nerve root injury	2 (3.9)	1 (1.8)	0
Cauda equina injury	0	0	0
Sympathetic chain injury	0	2 (3.6)	0
Transient thigh pain/numbness	6 (11.8)	1(1.8)	1 (2.0)
Transient iliolumbar weakness	2 (3.9)	1(1.8)	1 (2.0)
Cage sedimentation or shifting	6(11.8)	7(12.5)	5 (10.2)
Surgical Instrument failure	0	1 (1.8)	0
Intervertebral space infection	1 (2.0)	0	0
Pneumonia	1 (2.0)	0	1 (2.0)
Cardio-cerebrovascular events	0	1 (1.8)	0
Lower extremity deep vein thrombosis	0	0	0
Total	21	20	10

*XLIF* indicated extreme lateral interbody fusion, *OLIF* oblique lumbar interbody fusion

### Intraoperative complications

#### Vascular injury

In the OLIF group, there were 3 cases (5.4%) of vascular injury. One case was a segmental lumbar artery rupture and bleeding, and the electrode was used to stop the bleeding. One case of iliac vein was bleeding with large laceration, which was clearly exposed and sutured after extending the skin incision. One case of ascending lumbar vein hemorrhage was treated with hemostatic material and gauze. Compared with OLIF, the XOLIF and XLIF reduced vascular injury. There was no vascular injury in the XLIF and XOLIF groups.

#### Others

One case of peritoneal laceration occurred and was sutured immediately in OLIF group. Endplate injury occurred in all three groups, including 3 cases (5.9%) in the XLIF group, 2 cases (3.6%) in the OLIF group, and 2 cases (4.1%) in the XOLIF group.

### Postoperative complications

All patients got out of bed on the second day after surgery, and there was no occurrence of deep vein thrombosis in the lower limbs. There were no cases of postoperative intestinal obstruction, ureteral injury, or abdominal organ injury.

#### Nerve injury

**Fig. 6:**
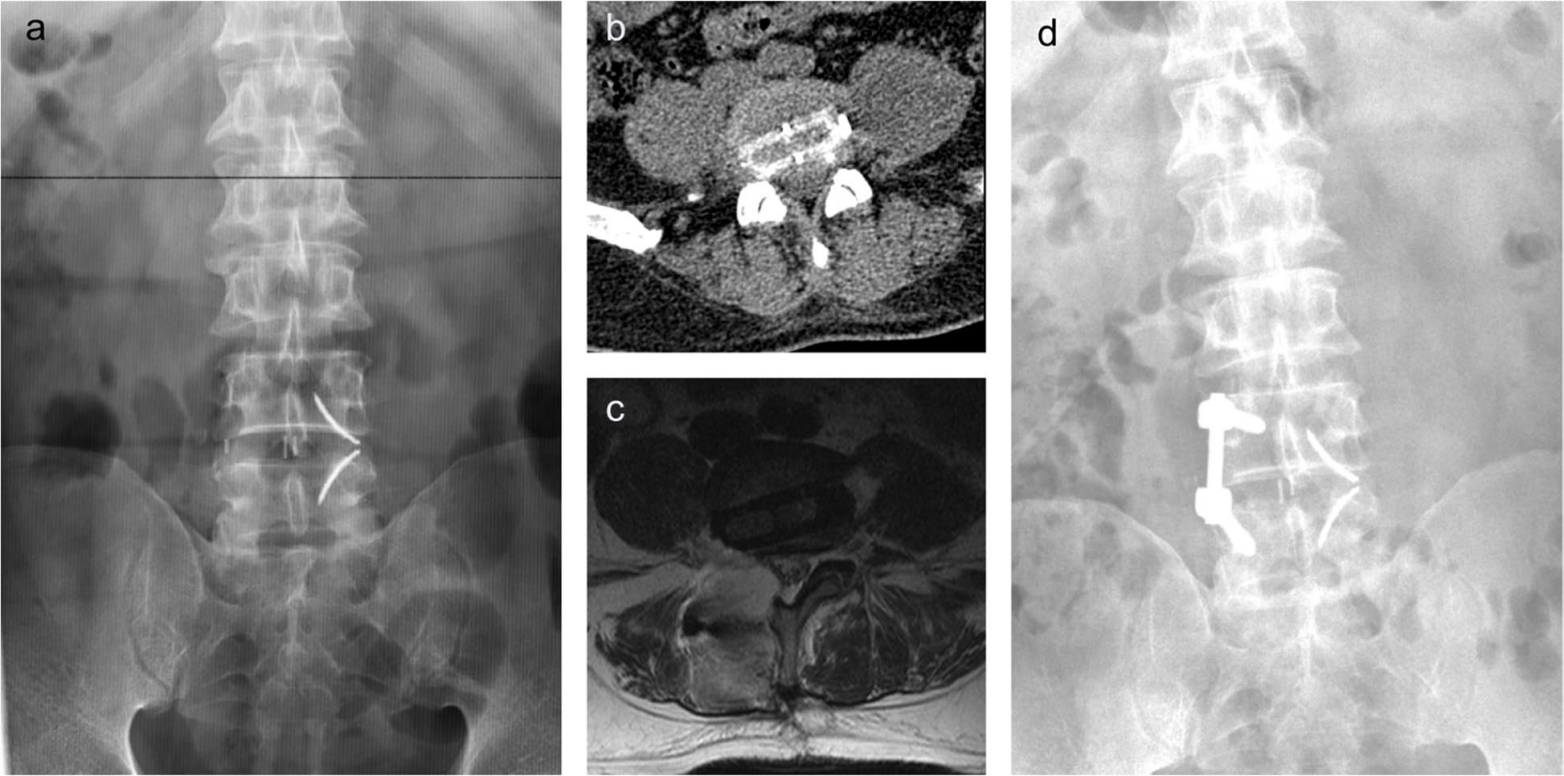
A case was revised with TLIF for contralateral nerve root injury. **a** Anteroposterior X-ray of lumbar spine after XLIF. **b** Axial CT showed that the right nerve root was compressed by the cage after XLIF. **c** Axial T2-weighted MRI image after revision indicated right decompression. **d** Anteroposterior X-ray of lumbar spine after revision

The contralateral nerve root was injured in 2 cases (3.9%) with psoas hypertrophy in the XLIF group. The contralateral nerve root was compressed by cage on postoperative CT. In one of the 2 cases, we re-adjusted the position of the cage through the original incision on the second postoperative day, and the pain in the lower limbs was relieved. The other one was revised with transforaminal lumbar interbody fusion (TLIF) on the right side with unilateral fixation, and radiating pain in the right leg was relieved after removing partial cage pressing on the right nerve root (Fig. 6). One case (1.8%) of contralateral nerve root injury caused by testing cage model improved due to timely intraoperative detection and adjustment. There were 2 cases of sympathetic chain injury in OLIF group. Fortunately, the symptoms disappeared during postoperative outpatient review. Transient pain and numbness in the front of the thigh or iliopsoas muscle weakness occurred in 8 cases (15.7%), 2 cases (3.6%) and 2 cases (4.1%) in XLIF group, OLIF group and XOLIF group, respectively, and there was a statistical difference among three groups (*P* = 0.029).

#### Cage sedimentation and instrument failure

Cage subsidence occurred in 6 cases (11.8%) in the XLIF group, 7 cases (12.5%) in the OLIF group, and 5 cases (10.2%) in the XOLIF group during follow-up. One case (1.8%) of lateral plate shedding occurred in the OLIF group.

#### Others

One case of postoperative intervertebral infection occurred in the XLIF group, which was cured after 2 weeks of antibiotic treatment. One case of pneumonia occurred in the XLIF group and 1 case in the XOLIF group, and recovered after drug treatment. One case of acute cerebral embolism occurred in the OLIF group after the operation, which was unable to identify whether it was related to surgery.

## Discussion

XLIF and OLIF are the two most commonly used LLIF for the treatment of lumbar spine disorders, which have been popular among spine surgeons since their introduction due to their minimally invasive nature, high fusion rate, and precise efficacy. XLIF splits the psoas major muscle to operate, and how to avoid lumbar plexus nerve injury is a constant concern among scholars [4, 5]. Intraoperative neurophysiological monitoring can effectively reduce lumbar plexus nerve injury, but it is a passive measure with low specificity and high cost, which is not suitable for primary hospitals. Based on the anatomical study of the lumbar plexus, the traditional XLIF was modified in our center in advance, and it was not necessary to be routinely equipped with neurophysiological monitoring intraoperatively [9]. Previously, we predominantly made modifications to the surgical entry point. However, the cage was still implanted vertically with the assistance of a 3-blade retractor, which required making the break of the table between the iliac crest and greater trochanter for side bending to eliminate iliac crest occlusion in the L45 segment with high iliac crest. During the later application, we found that series of problems including the tension of the psoas major muscle due to the break of the table, the mutual constraint of the forces between the three blades of the retractor and the inability to place the retractor vertically due to the high iliac crest could lead to the inability to implant the cage vertically.

OLIF was first reported by Mayer in 1997 and was officially named and designed with a dedicated two-blade retractor by Silvestre in 2012 [11, 12]. OLIF chooses an oblique approach between the psoas major muscle and the large artery without splitting the psoas major muscle, which is less likely to interfere with lumbar plexus, but is likely to cause injury to the sympathetic trunk, ureter, vessels, and peritoneum around the approach. Based on the narrower space between psoas major muscle and large artery in Asians compared to Europeans and Americans, Fan et al. [13] proposed a modified OLIF approach by separating the anterior border of the psoas major muscle dorsally from the disc and/or vertebral body under direct vision to reveal sufficient disc area. This modified OLIF approach reduced complications to some extent, and this study reported an access-related complication rate of 15.6% (13/83). Even so, this modified OLIF is mostly unsuitable in cases with extremely narrow space between psoas major muscle and artery, elevated psoas major muscle, and right-sided approach.

Based on the pre-modified XLIF, we proposed XOLIF by combining the advantages of OLIF tilted placement of 2-blade retractor. XOLIF differs from XLIF in the following ways: (1) the break of the table between the iliac crest and greater trochanter for side bending is not needed in the L45 segment with high iliac crest; (2) a 2-bladed retractor is used; (3) the retractor is placed at an oblique angle; and (4) the psoas major muscle is retracted dorsally using Kirschner wires. XOLIF has many technical advantages. (1) The 2-blade retractor is placed at an oblique angle, suitable for the high iliac crest L45 segment. (2) Splitting the psoas major muscle at the anterior edge of the psoas major muscle on direct vision for access reduces the risk of vascular, nerve, and organ injury, as well as lumbar plexus injury. (3) In the past, the OLIF procedure often required an assistant to use tissue pulling hooks to pull the psoas major dorsally, which was laborious and unstable, and the result was unsatisfactory when the psoas major was thickened. The dorsal muscle can easily herniate into the operating area and affect the operation with 3-blade retractor of XLIF. The use of 2 Kirschner wires as a retractor is easy and has the best effect in retracting psoas major muscle, solving the problem of blade occlusion during intraoperative fluoroscopy as well. (4) A 1.5 mm-diameter Kirschner wire with a guide rod is inserting into the center of the intervertebral space. The position of the apex of the guide rod is confirmed by lateral fluoroscopy to precisely guide the lateral disc incision position and to avoid implanting cage extremely ventrally or dorsally.

The present study showed that there was no statistical difference among the three groups in terms of operation time, intraoperative blood loss, postoperative VAS and ODI. Besides, the interbody fusion rate evaluated by CT scan at 12 months after surgery reached more than 90%, and the fusion rate of the three groups was not statistically different. Factors such as fusion cage material, the type of filler, bone graft material, internal fixation method, smoking, obesity and osteoporosis can affect interbody fusion rate [14–16]. A study by Nourian et al. [17] showed that BMP can improve LLIF interbody fusion rate. Internal fixation can create a good mechanical stability for interbody fusion. Adjunctive internal fixation is recommended for patients with osteoporosis, spondylolisthesis, and intraoperative endplate injury, but there are no specific criteria for the way of internal fixation [18, 19].

To our knowledge, this study was the first time to analyze the composition ratio of cases with psoas major and vascular space stenosis, psoas major muscle elevation, psoas major muscle hypertrophy, and high iliac crest in each group. The composition ratio of cases with psoas major and vascular space stenosis, psoas major muscle elevation was significantly higher in the XOLIF and XLIF groups than that in the OLIF group, because these two types are mostly unsuitable for OLIF and have a higher risk of vascular and nerve injury in the access [20]. The risk can be predicted preoperatively by assessing the condition of the space between psoas major and artery with lumbar MRI [21]. The L4-5 segment with high iliac crest cannot be performed with traditional XLIF because of the occlusion of the iliac crest. Incisions of XOLIF and OLIF are more ventral, and the 2-blade retractor is placed obliquely just to avoid the blocked iliac crest. As a result, the cage can be implanted vertically with a special angled handle [22]. Although hypertrophy of psoas major muscle is not contraindication for traditional XLIF, it is relatively difficult to operate intraoperatively. XOLIF does not make the break of the table between the iliac crest and greater trochanter for side bending during the procedure, which keeps the muscles in a relaxed state. In addition, it is easy to implant cage vertically by 2 Kirschner wires retracting psoas major muscle dorsally. XOLIF combines advantages of OLIF and XLIF in treatment of the above cases, with wider indications.

Perioperative complication is the key factor to evaluate the technique. The OLIF approach through the space between the psoas major muscle and the vessels avoids interference with the lumbar plexus, but the risk of injury to the sympathetic trunk, ureter, vessels, and peritoneum is higher compared to XLIF [23]. The XLIF approach with vertically splitting the psoas major muscle operates far away from the sympathetic trunk, ureter, vessels, and peritoneum, but the risk of injury to the lumbar plexus is higher. Differences of approaches between OLIF and XLIF lead to differences in access-related complications [24]. XOLIF takes both advantages into account in terms of surgical safety.

Silvestre et al. [12] reported an overall complication rate of 11.2% in 179 cases of OLIF surgery. The access-related complications included 2 cases of rupture of iliac vein, 1 case of rupture of iliac-lumbar vein, 1 case of rupture of peritoneum, and 3 cases of sympathetic chain injury, 2 cases of neurological deficit, 2 cases of psoas muscle weakness or thigh numbness. Shunsuke et al. [25] retrospectively investigated a total of 2998 cases of minimally invasive LLIF performed from 2013 to 2015, including 1995 cases of XLIF and 1003 cases of OLIF, with an overall complication rate of 18% for XLIF 19.4% and OLIF 15.3%. However, complications such as intraoperative endplate injury and cage subsidence were not counted in the above studies, which may account for the low overall rate. Abe et al. [26] conducted a retrospective study of 155 cases of OLIF carried out by multiple centers. The overall incidence of complications was 48.3% (75/155). The three most common complications were endplate fractures or cage subsidence (18.7%), transient iliopsoas muscle weakness or thigh numbness (13.5%), and segmental artery injury (2.6%).

A total of 51 (32.7%) intraoperative and postoperative complications occurred in 156 patients in the present study, and most of these were minor complications including 7 cases (4.5%) of intraoperative endplate injury, 18 cases (11.5%) of postoperative cage subsidence, and 12 cases (7.7%) of transient iliopsoas muscle weakness or thigh pain/numbness. The incidence of endplate injury and cage subsidence was not higher in the three groups of this study than that of previous studies. The key to preventing endplate injury is to operate gently with parallel gaps, not to use reamers excessively, and select the appropriate cage. Cage subsidence is affected by many factors, including obesity, osteoporosis, intraoperative endplate injury, oversized cage, internal fixation method, etc. [27, 28]. Preoperative identification of risk factors for cage subsidence, intraoperative non-injury to the endplate and selection of an appropriate internal fixation method are the keys to preventing postoperative cage subsidence. In this study, 3 cases of vascular injury, 2 cases of sympathetic chain injury, and 1 case of peritoneal rupture occurred in the OLIF group, whereas, no such complications occurred intraoperatively in the XOLIF and XLIF groups because the access was far from great vessels, sympathetic chain, and peritoneum. Postoperative transient iliopsoas muscle weakness or thigh pain/numbness was often seen after LLIF, mostly due to intraoperative injury or irritation of psoas major muscle and psoas plexus [4, 29]. Because OLIF does not split the psoas major muscle, the incidence of postoperative iliopsoas muscle weakness and thigh paresthesia is lower than that of XLIF [29]. XOLIF splits only a portion of the anterior border muscle fibers of the psoas major, which can take into account the advantage of OLIF not to interfere too much with the psoas major.

There were some limitations of this study. Firstly, the study just included L4-5 cases. Of course, XOLIF as well as XLIF and OLIF can be used in segments above L45. However, the upper lumbar segments do not have conditions of psoas major and vascular space stenosis, psoas major muscle elevation, psoas major muscle hypertrophy, and high iliac crest, which cannot highlight the advantages of XOLIF, so the study did not include segments above L45. Secondly, the study was a single-center retrospective cohort study, and future multicenter prospective studies are needed to further validate the superiority of XOLIF.

## Conclusions

XOLIF has made a series of improvements to XLIF in terms of the utilization of retractor and the process of operation, taking advantages of XLIF and OLIF into account, with a wider range of indications. XOLIF has shown perfect clinical efficacy in the treatment of lumbar degenerative diseases with a low incidence of intraoperative and postoperative complications, especially in cases of psoas major and vascular space stenosis, psoas major muscle elevation, psoas major muscle hypertrophy, and high iliac crest, which is worth promoting.

## Data Availability

The datasets used and/or analysed during the current study are available from the corresponding author on reasonable request.

## Abbreviations

XLIF: Extreme lateral interbody fusion
OLIF: Oblique lumbar interbody fusion
LLIF: Lateral lumbar interbody fusion
BMI: Body mass index
VAS: Visual analogue scale
ODI: Oswestry disability index
BMP: Bone morphogenetic protein
TLIF: Transforaminal lumbar interbody fusion
